# Association of dietary intakes of vitamin B12, vitamin B6, folate, and methionine with the risk of esophageal cancer: the Japan Public Health Center-based (JPHC) prospective study

**DOI:** 10.1186/s12885-021-08721-8

**Published:** 2021-09-01

**Authors:** Paramita Khairan, Tomotaka Sobue, Ehab Salah Eshak, Ling Zha, Tetsuhisa Kitamura, Norie Sawada, Motoki Iwasaki, Manami Inoue, Taiki Yamaji, Taichi Shimazu, Hiroyasu Iso, Shoichiro Tsugane

**Affiliations:** 1 Department of Social and Environmental Medicine, Graduate School of Medicine, Osaka University, 2-2 Yamadaoka, Suita, Osaka 565-0871 Japan; 2 Department of Internal Medicine, Faculty of Medicine, University of Muhammadiyah, Jakarta, Indonesia; 3 Public Health, Department of Social Medicine, Osaka University Graduate School of Medicine, Suita, Osaka 565-0871 Japan; 4 Department of Public Health and Preventive Medicine, Faculty of Medicine, Minia University, Minya, Egypt; 5 Epidemiology and Prevention Group, Center for Public Health Sciences, National Cancer Center, Chuo-ku Tokyo, 104-0045 Japan

**Keywords:** Esophageal cancer, One-carbon metabolism, Vitamin B12 intake, Folate intake, Prospective cohort study

## Abstract

**Background:**

B vitamins and methionine are essential substrates in the one-carbon metabolism pathway involved in DNA synthesis and methylation. They may have essential roles in cancer development. We aimed to evaluate the associations of dietary intakes of vitamin B12, vitamin B6, folate, and methionine with the risk of esophageal cancer (EC) using data from the Japan Public Health Center-based Prospective Study.

**Methods:**

We included 87,053 Japanese individuals who completed a food frequency questionnaire and were followed up from 1995–1998 to 2013 and 2015. Hazard ratios (HRs) and 95% confidence intervals (CIs) were calculated by Cox proportional-hazard regression across quintiles of dietary intakes of B vitamins and methionine.

**Results:**

After 1,456,678 person-years of follow-up, 427 EC cases were documented. The multivariable HR (95% CI) of incident EC in the highest versus lowest quintile of dietary intake of vitamin B12 was 1.75 (1.13–2.71; *p-*trend=0.01). Stratification analysis based on alcohol consumption showed that higher dietary intakes of vitamin B12 and methionine were associated with an increased risk of EC among never-drinkers; HRs (95% CI**s**) were 2.82 (1.18–6.74; *p*-trend=0.009; *p-*interaction=0.18) and 3.45 (1.32–9.06; *p*-trend=0.003; *p*-interaction 0.02) for vitamin B12 and methionine, respectively. Meanwhile, there was no association between vitamin B12 and methionine intake with the risk of EC among drinkers. There were no associations between dietary intake of folate or vitamin B6 and the risk of EC.

**Conclusion:**

Dietary intake of vitamin B12 was positively associated with the risk of EC in the Japanese population.

**Supplementary Information:**

The online version contains supplementary material available at 10.1186/s12885-021-08721-8.

## Background

Esophageal cancer (EC) is the sixth most common cause of cancer-related mortality worldwide, particularly in eastern Asian and eastern African regions [1]. Esophageal squamous cell carcinomas account for 88% of EC cases, with an increasing trend of incidence among women in several countries, including Japan [2].

Lifestyle habits such as alcohol consumption and tobacco smoking are the main risk factors for esophageal squamous cell carcinoma [3]. It has been suggested that diets rich in foods of animal origin and low in foods containing vitamins C and E are associated with an increased risk of EC [4]. Specifically, the total intake of red meat (supposedly high in vitamin B12) has been positively associated with an increased risk of EC [5, 6].

Vitamin B12 and folate are the primary components of the one-carbon metabolism (OCM) pathway involved in DNA synthesis, repair, and methylation [7, 8]. Additionally, vitamin B6 and methionine are also co-factors that are required in the OCM pathway [9]. A dietary imbalance or deficiency in those nutrients may disrupt DNA methylation or induce the disincorporation of nucleotide synthesis, which could lead to carcinogenesis [9].

Recently, the dietary intakes of folate and vitamin B12 were shown to be associated with the risk of several cancers, such as colorectal, breast, pancreatic, and lung cancers [10–13]. The associations of folate and vitamin B12 intakes with EC risk have been inconsistent, and most of the studies that investigated these associations did not utilize a prospective design [14–19]. Two previous case-control studies that investigated the association between EC risk and dietary folate intake reported reduced EC risk, even among smokers and alcohol drinkers [15, 16]. Alcohol drinking, a strong risk factor for EC, may modify the association between dietary intakes of B vitamins and the risk of EC because alcohol affects the absorption and metabolism of vitamin B12 and folate [20, 21].

Herein, we conducted a prospective cohort study in a large Japanese population to clarify the association of dietary intake of vitamin B12, vitamin B6, folate, and methionine with the risk of EC and to investigate the hypothesized potential effect modifications by alcohol consumption.

## Methods

### Study design and participants

The Japan Public Health Center-based Prospective Study (JPHC Study) is a large population-based cohort study that began in 1990–1994 to investigate the risk factors for cancer, metabolic diseases, and other lifestyle-related diseases. Complete details of the study design are available elsewhere [22]. This study was approved by the Institutional Review Board of the National Cancer Center, Japan, and Osaka University.

**Fig. 1:**
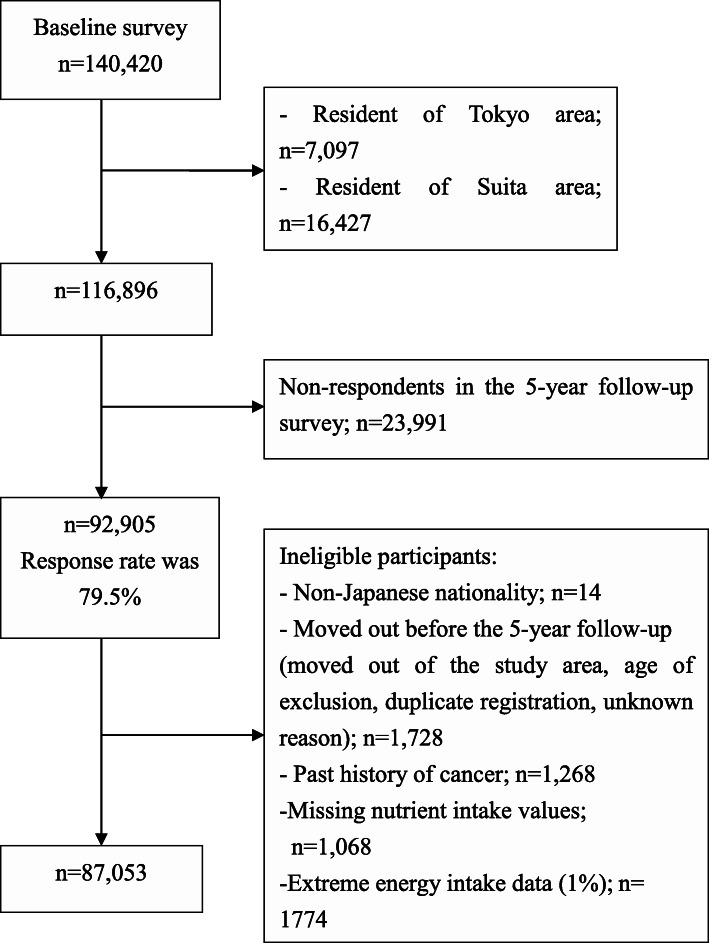
Flow chart of the study participants involved in the 5-year follow-up survey (baseline of the current study)

The participants eligible for this study were those who participated in the JPHC study who gave feedback in the 5-year follow-up self-administered questionnaire surveys. The 5-year follow-up self-administered questionnaire surveys was including more comprehensive information of food intake’s frequency. The surveys were distributed to residents of 11 public health center areas in 1995–1999 who were aged 45–74 years. Participants registered in Tokyo and Suita areas were excluded because of information’s unavailability on cancer incidence in Tokyo area and a different study populations’ definition in Suita area. We excluded participants who met one or more of the following exclusion criteria: diagnosis of EC during the period between the baseline survey administration and 5-year follow-up survey (*n*=18), history of cancer (*n*=1,250), missing values for the nutrients evaluated in the study (*n*=1,068), or excessive energy intake (subjects who reported energy intake in the upper or lower 1 % of intake [*n*=1,774]). Finally, data of a total of 87,053 men and women were eligible for the analysis. Details are shown in the flow chart of the study participants (Fig. 1).

### Assessment of exposures and covariates

The 5-year follow-up questionnaire survey included a food frequency questionnaire (FFQ) to estimate the dietary intake of 147 food items. Responses of diet frequency for each food item ranged from “rarely,” “1–3 days/month,” “1–2 days/week,” “3–4 days/week,” or “5–6 days/week,” to “once/day,” “2–3 times/day,” “4–6 times/day,” or “≥7 times/day.” Energy-adjusted dietary intakes of vitamin B12, vitamin B6, folate, and methionine were calculated using the residual method, and daily nutrient intake was calculated using the Standard Table of Food consumption in Japan, 7^th^ revised and enlarged edition [23, 24]. The supplemental nutrition intake was not included in the current analysis because of the lack of a database of the supplements. The validity of vitamin B12, vitamin B6, folate, and methionine estimated from the FFQ was evaluated from a subsample of participants in the JPHC Study Cohorts I and II [25, 26]. Regarding the validity of the FFQ, energy-adjusted Spearman's correlation coefficients between intake values of vitamin B12, vitamin B6, and folate derived from the FFQ and those derived from the 28-day or 14-day dietary records in men were 0.33, 0.45, and 0.40 in Cohort I, respectively, and 0.35, 0.36, 0.50 in Cohort II, respectively. For women, the correlation coefficients were 0.34, 0.47, and 0.35 in Cohort I, respectively, and 0.27, 0.40, and 0.48 in Cohort II, respectively [26]. Regarding the validity of the FFQ for methionine intake, Spearman's correlation coefficients were 0.29 and 0.27 for men and women, respectively, in Cohort I. In Cohort II, the values were 0.27 and 0.31 for men and women, respectively [25]. The questionnaire also asked the participants to report their demographic characteristics, medical history, smoking and drinking habits, lifestyle, occupation, height and weight, physical activity, working hours, stress, and other life habits.

### Ascertainment of EC cases

EC data were collected from the cancer registries, medical records from hospitals in the study areas, reviews of the death certificate, or both medical records and death certificates. We identified the cases using the International Classification of Disease for Oncology Codes, third edition code “C15” with histology code 8050–8084 [27].

### Statistical analyses

Statistical analyses were based on EC incidence values during 15.8 median years of follow-up until December 31, 2013 for Kochi and Nagasaki area, and December 31, 2015 for the other PHC areas. For each individual, person-years of follow-up were calculated from the day of submitting the 5-year follow-up survey to whichever of these endpoints occurred first: diagnosis of EC, death, emigration, or December 31, 2013. The hazard ratios (HRs) with 95% confidence intervals (CIs) of the incidence of EC were calculated using time-dependent Cox proportional hazard models according to the quintiles of dietary intakes of vitamin B12, vitamin B6, folate, and methionine after adjusting for age (5-year categories), sex, and study area in Model 1. Model 2 was additionally adjusted for quintiles of the body mass index in kg/m^2^, physical activity in metabolic equivalent of task unit (quintiles), alcohol consumption (not current drinker, current drinker of ≤150 g/week, 151–≤300 g/week, 301–<450 g/week, ≥450 g/week, or missing), smoking (never, former, current smoker of ≤19 cigarettes/day, 20–29 cigarettes/day, ≥30 cigarettes/day, or missing), and family history of cancer (yes or no). Model 3 was adjusted further for vitamin B12, vitamin B6, folate, and methionine mutually to estimate the independent effect of the nutrients. Sensitivity analysis was conducted by excluding EC cases diagnosed within three years after the study’s enrolment. We also performed tests for linear trends across the intake quintile categories of the studied B vitamins and methionine by adding to the model and testing the significance of variables representing each category's median values.

As alcohol is a strong risk factor for EC and affects the absorption and metabolism of B vitamins [20, 21], and as Japanese men are more likely to drink than women [28], we conducted stratified analyses based on alcohol intake status (never-drinker, low-drinker/alcohol intake 0 -150 g/week, and high-drinker/alcohol intake> 150 g/week) in all participants and men. We attained the *p*-trend for each level of alcohol intake and *p*-interaction for a cross-product term of the alcohol categories and rank variables for quintiles of dietary intakes of B vitamins and methionine. All statistical analyses were two-tailed, with *p*<0.05 as the statistically significant level, and were conducted using SAS software (version 9.4; SAS Institute).

## Results

Table 1 shows the distribution of EC risk factors across the quintiles of dietary intake of vitamin B12, vitamin B6, folate, and methionine. The participants in the highest quintile of dietary intakes of these nutrients tended to be slightly older, consumed less alcohol, and were less likely to smoke (except for the high smoking prevalence in the highest quintile of dietary vitamin B12 intake).

**Table 1:** Baseline characteristics of the participants according to quintiles of energy-adjusted dietary intakes of vitamin B12, vitamin B6, folate, and methionine

Characteristics	Q1	Q2	Q3	Q4	Q5	***p*** value^**a**^
**Vitamin B12 **^**b**^						
No. of participants	17,410	17,411	17,411	17,411	17,410	
Male, n (%)	8,003 (46.0)	7,555 (43.4)	7,978 (45.8)	8,290 (47.6)	8,890 (51.1)	<.0001
Age, years ^c^	56.2 ± 7.9	56.7 ± 8.0	56.8 ± 8.0	57.2 ± 7.7	57.7 ± 7.5	<.0001
BMI, kg/m^2 c^	23.8 ± 3.5	23.7 ± 3.5	23.5 ± 3.2	23.5 ± 3.3	23.5 ± 3.4	<.0001
Current smokers, n (%)	3,877 (22.3)	3,672 (21.1)	3,783 (21.7)	3,927 (22.6)	4,396 (25.3)	<.0001
Family history of cancer, yes (n [%])	2,249 (12.9)	2,454 (14.1)	2,627 (15.1)	2,829 (16.3)	2,828 (16.2)	<.0001
METs unit ^c^	34.2 ± 6.3	33.5 ± 6.0	33.2 ± 5.9	33.3 ± 6.0	33.6 ± 6.1	<.0001
Alcohol consumption, ethanol g/week ^c^	163.9 ± 299.4	94.8 ± 180.3	84.6 ± 163.8	85.8 ± 162.5	102.7 ± 180.7	<.0001
Alcohol, n (%)						0.004
Non-drinkers	9,333 (53.6)	9,956 (57.2)	9,899 (56.9)	9,591 (55.1)	9,224 (53.0)	
≤150 g/week	2,554 (14.7)	3,139 (18.0)	3,425 (19.7)	3,695 (21.2)	3,380 (19.4)	
150<alcohol≤300 g/week	1,282 (7.4)	1,497 (8.6)	1,646 (9.5)	1,672 (9.6)	1,827 (10.5)	
300<alcohol<450 g/week	1,260 (7.2)	1,158 (6.7)	1,107 (6.4)	1,134 (6.5)	1,302 (7.5)	
≥450 g/week	2,619 (15.0)	1,226 (7.0)	912 (5.2)	917 (5.3)	1,243 (7.1)	
Energy, kcal/day ^c^	2,398.1 ± 710.5	1,859.0 ± 572.3	1,774.0 ± 602.0	1,854.9 ± 627.3	2,218.3 ± 746.4	<.0001
Vitamin B6, mg/day ^c^	1.3 ± 0.4	1.4 ± 0.2	1.5 ± 0.2	1.6 ± 0.2	1.9 ± 0.4	<.0001
Vitamin B12, μg/day ^c^	3.7 ± 2.6	7.4 ± 0.6	9.3 ± 0.6	11.6 ± 0.8	18.3 ± 6.7	<.0001
Folate, μg/day ^c^	361.8 ± 200.0	392.5 ± 135.3	406.5 ± 127.7	425.6 ± 132.1	453.7 ± 166.4	<.0001
Methionine, mg/day ^c^	1,293.0 ± 363.3	1,525.3 ± 215.1	1,638.0 ± 211.3	1,763.6 ± 230.0	2,079.1 ± 469.0	<.0001
**Vitamin B6** ^**b**^						
No. of participants	17,410	17,411	17,411	17,411	17,410	
Male, n (%)	6,848 (39.3)	6,429 (36.9)	7,768 (44.6)	9,091 (52.2)	10,580 (60.8)	<.0001
Age, years ^c^	55.0 ± 7.6	56.3 ± 7.9	57.2 ± 7.9	57.7 ± 7.7	58.5 ± 7.6	<.0001
BMI, kg/m^2c^	23.7 ± 3.4	23.5 ± 3.3	23.5 ± 3.3	23.6 ± 3.5	23.7 ± 3.4	0.05
Current smokers, n (%)	4,237 (24.3)	3,511 (20.2)	3,654 (21.0)	3,986 (22.9)	4,268 (24.5)	<.0001
Family history of cancer, yes (n [%])	2,191 (12.6)	2,423 (13.9)	2,673 (15.4)	2,744 (15.8)	2,956 (17.0)	<.0001
METs unit ^c^	33.8 ± 6.2	33.2 ± 5.9	33.4 ± 6.0	33.5 ± 6.1	33.8 ± 6.2	<.0001
Alcohol consumption, ethanol g/week ^c^	151.1 ± 296.8	80.5 ± 172.6	80.8 ± 163.6	95.1 ± 167.8	124.1 ± 188.7	<.0001
Alcohol, n (%)						<.0001
Non-drinkers	9,988 (57.4)	1,0644 (61.1)	10,082 (57.9)	9,151 (52.6)	8,135 (46.7)	
≤150 g/week	2,627 (15.1)	3,104 (17.8)	3,494 (20.1)	3,621 (20.8)	3,347 (19.2)	
150<alcohol≤300 g/week	1,060 (6.1)	1,269 (7.3)	1,465 (8.4)	1,916 (11.0)	2,214 (12.7)	
300<alcohol<450 g/week	824 (4.7)	912 (5.2)	1,043 (6.0)	1,338 (7.7)	1,845 (10.6)	
≥450 g/week	2,590 (14.9)	1,031 (5.9)	875 (5.0)	984 (5.7)	1,434 (8.2)	
Energy, kcal/day ^c^	2,265.4 ± 735.6	1,833.1 ± 615.5	1,817.3 ± 609.6	1,917.5 ± 623.3	2,270.8 ± 742.7	<.0001
Vitamin B6, mg/day ^c^	1.1 ± 0.2	1.4 ± 0.04	1.5 ± 0.04	1.7 ± 0.1	2.1 ± 0.3	<.0001
Vitamin B12, μg/day ^c^	5.7 ± 4.0	8.5 ± 3.2	9.7 ± 3.5	11.2 ± 4.2	15.0 ± 8.2	<.0001
Folate, μg/day ^c^	280.8 ± 109.9	364.3 ± 94.4	405.6 ± 102.8	449.7 ± 121.3	539.6 ± 202.7	<.0001
Methionine, mg/day ^c^	1,377.7 ± 371.2	1,561.9 ± 242.1	1,644.6 ± 252.9	1,736.3 ± 286.3	1,978.2 ± 544.5	<.0001
**Folate** ^**b**^						
No. of participants	17,410	17,411	17,411	17,411	17,410	<.0001
Male, n (%)	10,072 (57.9)	8,732 (50.2)	7,646 (43.9)	7,310 (42.0)	6,956 (40.0)	<.0001
Age, years ^c^	54.9 ± 7.5	56.3 ± 7.9	57.0 ± 7.9	57.8 ± 7.8	58.7 ± 7.7	<.0001
BMI, kg/m^2 c^	23.7 ± 3.3	23.6 ± 3.4	23.5 ± 3.3	23.6 ± 3.4	23.6 ± 3.5	0.03
Current smokers, n (%)	5,664 (32.5)	4,148 (23.8)	3,557 (20.4)	3,254 (18.7)	3,033 (17.4)	<.0001
Family history of cancer, yes (n [%])	2,222 (12.8)	2,343 (13.5)	2,650 (15.2)	2,814 (16.2)	2,958 (17.0)	<.0001
METs unit ^c^	33.9 ± 6.4	33.3 ± 6.1	33.3 ± 6.0	33.6 ± 6.0	33.7 ± 6.0	0.004
Alcohol consumption, ethanol g/week ^c^	209.3 ± 311.1	103.7 ± 182.4	82.0 ± 159.2	72.6 ± 150.4	63.4 ± 140.6	<.0001
Alcohol intake, n (%)						<.0001
Non-drinkers	7,560 (43.4)	9,272 (53.3)	9,943 (57.1)	10,401 (59.7)	10,819 (62.1)	
≤150 g/week	2,661 (15.3)	3,338 (19.2)	3,452 (19.8)	3,397 (19.8)	3,345 (19.2)	
150<alcohol≤300 g/week	1,695 (9.7)	1,797 (10.3)	1,600 (9.2)	1,490 (8.6)	1,342 (7.7)	
300<alcohol<450 g/week	1,701 (9.8)	1,346 (7.7)	1,087 (6.2)	956 (5.5)	871 (5.0)	
≥450 g/week	3,496 (20.1)	1,247 (7.2)	861 (5.0)	737 (4.2)	576 (3.3)	
Energy, kcal/day ^c^	2,335.4 ± 727.8	1,880.2 ± 634.5	1,837.6 ± 622.5	1,910.2 ± 645.3	2,140.9 ± 725.3	<.0001
Vitamin B6, mg/day ^c^	1.3 ± 0.4	1.5 ± 0.3	1.6 ± 0.3	1.6 ± 0.3	1.8 ± 0.4	<.0001
Vitamin B12, μg/day ^c^	8.0 ± 6.7	9.7 ± 4.7	10.4 ± 4.9	10.8 ± 5.0	11.4 ± 6.9	<.0001
Folate, μg/day ^c^	224.5 ± 68.2	326.7 ± 18.4	388.1 ± 18.0	461.6 ± 26.1	639.2 ± 146.7	<.0001
Methionine, mg/day ^c^	1,547.7 ± 525.4	1,655.1 ± 346.1	1,691.4 ± 342.1	1,706.8 ± 348.6	1,698.1 ± 428.7	<.0001
**Methionine** ^**b**^						
No. of participants	17,410	17,411	17,411	17,411	17,410	
Male, n (%)	7,924 (45.5)	6,273 (36.0)	7,211 (41.4)	8,748 (50.2)	10,560 (60.7)	<.0001
Age, years ^c^	55.7 ± 7.8	56.7 ± 7.9	57.1 ± 8.0	57.3 ± 7.8	57.8 ± 7.6	<.0001
BMI, kg/m^2 c^	23.6 ± 3.4	23.6 ± 3.5	23.6 ± 3.4	23.6 ± 3.3	23.6 ± 3.3	0.06
Current-smokers, n (%)	4,620 (26.5)	3,364 (19.3)	3,439 (19.8)	3,811 (21.9)	4,422 (25.4)	<.0001
Family history of cancer, yes (n [%])	2,460 (14.1)	2,519 (14.5)	2,667 (15.3)	2,649 (15.2)	2,692 (15.5)	0.006
METs unit ^c^	34.2± 6.3	33.5 ± 6.0	33.3 ± 5.9	33.2 ± 6.0	33.4± 6.1	<.0001
Alcohol intake, ethanol g/week ^c^	221.4 ± 329.4	90.4± 167.2	71.8 ± 139.4	67.0 ± 131.4	80.8 ± 148.2	<.0001
Alcohol intake, n (%)						<.0001
Non-drinkers	8,214 (47.2)	10,251 (58.9)	10,241 (58.8)	9,929 (57.0)	9,368 (53.8)	
≤150 g/week	2,031 (11.7)	2,743 (15.8)	3,445 (19.8)	4,001 (23.0)	3,978 (22.9)	
150<alcohol≤300 g/week	1,249 (7.2)	1,532 (8.8)	1,635 (9.4)	1,665 (9.6)	1,843 (10.6)	
300<alcohol<450 g/week	1,692 (9.7)	1,326 (7.6)	1,023 (5.9)	881 (5.1)	1,039 (6.0)	
≥450 g/week	3,911 (22.5)	1,100 (6.3)	654 (3.8)	521 (3.0)	731 (4.2)	
Energy, kcal/day ^c^	2,323.2 ± 701.9	1,859.0 ± 594.3	1,799.7 ± 602.0	1,866.1 ± 622.7	2,256.3 ± 774.2	<.0001
Vitamin B6, mg/day ^c^	1.3 ± 0.4	1.5 ± 0.2	1.5 ± 0.2	1.6 ± 0.2	1.9 ± 0.4	<.0001
Vitamin B12, μg/day ^c^	5.0 ± 3.7	8.0 ± 2.7	9.6 ± 2.8	11.4 ± 3.5	16.2 ± 7.9	<.0001
Folate, μg /day ^c^	378.4 ± 202.1	401.1 ± 141.2	410.2 ± 130.6	420.0 ± 134.4	430.4 ± 164.4	<.0001
Methionine, mg/day ^c^	1,169.7 ± 247.7	1,485.1 ± 47.3	1,633.3 ± 41.6	1,797.1 ± 56.3	2,213.9 ± 411.9	<.0001

^a^ Chi-square test for qualitative variables, ANOVA for continuous variables.

^b^ Cut-offs for quintiles of dietary intake of vitamin B12 were 6.24, 8.35, 10.28, and 13.2μg /day. Cut-offs for quintiles of dietary intake of vitamin B6 were 1.31, 1.46, 1.61, and 1.79 mg/day. Cut-offs for quintiles of dietary intake of folate were 293.24, 357.78, 420.28, and 511.80μg /day. Cut-offs for quintiles of dietary intake of methionine were 1395.84, 1562.41, 1707.36, and 1904.13 mg/day.

^c^ Mean ± standard deviation, all such variables

During 1,456, 678 person-years of follow-up of 87,053 Japanese men and women aged 45–74 years, we documented 427 incident cases of EC (382 men and 45 women) comprising 332 esophageal squamous cell carcinomas, 17 esophageal adenocarcinomas, and 78 non-specific EC.

Table 2 presents the HRs of EC according to dietary intake of vitamin B12, vitamin B6, folate, and methionine. The dietary intake of vitamin B12 showed a positive association with the risk of EC (Model 2; HR and 95% CI in the highest versus lowest quintiles was 1.37 [1.00–1.86]; *p*-trend=0.04) (Table 2). The positive association between vitamin B12 intake and risk of EC became stronger after further adjustment for vitamin B6, folate, and methionine (Model 3; HR and 95% CI: 1.75 [1.13-2.71]; *p-*trend =0.01). After excluding EC cases diagnosed within three years of enrolment, the association did not alter materially (Model 3: HR and 95% CI: 1.74[1.09–2.78]; p-trend =0.02). No associations were observed between the dietary intakes of vitamin B6, folate, and methionine and risk of EC (Table 2).

**Table 2:** Hazard ratios (95% confident intervals) of esophageal cancer according to quintiles of energy-adjusted dietary intakes of vitamin B12, vitamin B6, folate, and methionine

	Q1	Q2	Q3	Q4	Q5	***p***-trend^**a**^
**Vitamin B12**						
Person-years	296,280	292,419	290,662	289,062	288,256	
Cases, n	80	72	82	87	106	
Model 1^b^	1.00 (reference)	0.93 (0.68–1.28)	1.00 (0.73–1.37)	1.00 (0.73–1.37)	1.13 (0.83–1.52)	0.31
Model 2^c^	1.00 (reference)	1.09 (0.79–1.51)	1.25 (0.91–1.72)	1.28 (0.93–1.76)	1.37 (1.00–1.86)	0.04
Model 3^d^	1.00 (reference)	1.13 (0.80–1.59)	1.43 (0.99–2.06)	1.58 (1.07–2.34)	1.75 (1.13–2.71)	0.01
*3-year exclusion analysis*						
Cases, n	71	64	71	79	91	
Model 3^d^	1.00 (reference)	1.13 (0.79–1.63)	1.40 (0.95–2.06)	1.64 (1.08–2.48)	1.74 (1.09–2.78)	0.02
**Vitamin B6**						
Person-years	296,267	294,533	291,293	288,886	285,698	
Cases, n	75	78	82	86	106	
Model 1^b^	1.00 (reference)	1.01 (0.73–1.39)	0.87 (0.63–1.19)	0.78 (0.57–1.07)	0.81 (0.60–1.09)	0.08
Model 2^c^	1.00 (reference)	1.24 (0.90–1.71)	1.16 (0.84–1.60)	1.08 (0.78–1.49)	1.08 (0.79–1.48)	0.98
Model 3^d^	1.00 (reference)	1.17 (0.83–1.64)	1.04 (0.72–1.48)	0.89 (0.61–1.30)	0.81 (0.53–1.24)	0.16
*3-year exclusion analysis*						
Cases, n	65	70	73	75	93	
Model 3^d^	1.00 (reference)	1.22 (0.85–1.76)	1.09 (0.74–1.60)	0.94 (0.63–1.42)	0.89 (0.57–1.40)	0.36
**Folate**						
Person-years	292,275	290,461	290,196	292,381	291,364	
Cases, n	107	83	79	89	69	
Model 1^b^	1.00 (reference)	0.80 (0.60–1.07)	0.81 (0.60–1.08)	0.88 (0.66–1.17)	0.67 (0.49–0.92)	0.03
Model 2^c^	1.00 (reference)	1.02 (0.76–1.37)	1.10 (0.82–1.49)	1.24 (0.93–1.67)	1.00 (0.72–1.37)	0.71
Model 3^d^	1.00 (reference)	1.01 (0.74–1.36)	1.10 (0.80–1.51)	1.27 (0.92–1.75)	1.07 (0.74–1.55)	0.50
*3-year exclusion analysis*						
Cases, n	95	71	76	74	60	
Model 3^d^	1.00 (reference)	0.97 (0.70–1.34)	1.19 (0.85–1.65)	1.18 (0.84–1.68)	1.04 (0.70–1.54)	0.65
**Methionine**						
Person-years	293,843	294,206	291,479	289,686	287,464	
Cases, n	107	92	53	74	101	
Model 1^b^	1.00 (reference)	0.98 (0.74–1.29)	0.51 (0.37–0.71)	0.60 (0.45–0.81)	0.69 (0.53–0.91)	0.001
Model 2^c^	1.00 (reference)	1.29 (0.97–1.72)	0.77 (0.55–1.09)	1.02 (0.74–1.40)	1.19 (0.88–1.60)	0.46
Model 3^d^	1.00 (reference)	1.10 (0.80–1.50)	0.61 (0.41–0.90)	0.77 (0.52–1.15)	0.91 (0.59–1.40)	0.53
*3-year exclusion analysis*						
Cases, n	96	81	48	66	85	
Model 3^d^	1.00 (reference)	1.08 (0.77–1.51)	0.62 (0.41–0.93)	0.76 (0.50–1.15)	0.83 (0.53–1.31)	0.34

^a^ Median values of vitamin B12, vitamin B6, folate, and methionine intakes in each quintile were used to test for a linear trend across .

^b^ Model 1 was adjusted for age, sex, and public health center area.

^c^ Model 2 was adjusted for age, sex, public health center area, body mass index (quintiles), smoking (never, past, current; ≤19 cigarettes/day, 20–29 cigarettes/day, or ≥30 cigarettes/day), alcohol consumption (non-drinkers, ≤150 g/week, 150<alcohol≤300 g/week, 300<alcohol<450 g/week, or ≤450 g/week), family history of cancer, and physical activity in METs (quintiles).

^d^ Model 3 was model 2 that was mutually adjusted further for vitamin B12, vitamin B6, folate, and methionine

In the associations of the dietary intakes of both vitamin B12 and methionine with the risk of EC, alcohol intake confounded the associations. The adjustment for alcohol intake contributed to the increase in EC risk estimates across the dietary intake quintiles of vitamin B12 and disappearance of the inverse association observed for folate and methionine. As alcohol consumption differs greatly according to sex, we tested the sex-specific associations of dietary intakes of vitamins B12, folate, and methionine with the risk of EC and found no significant sex-specific interactions (*p*>0.1). The sex-specific associations did not reach a level of significance in either sex due to the loss of power (data not shown in table).

We conducted stratified analyses based on alcohol intake status for all participants (Table 3) and for men (Supplementary Table 1). The significant positive association between dietary intake of vitamin B12 and the risk of EC was only evident for participants who never consumed alcohol; however, the p-interaction did not reach the level of significance (*p*=0.18). A similar positive association was found for higher methionine intake in never drinkers, and the interaction with alcohol reached the level of statistical significance (*p*=0.02). The multivariable HRs (95% CIs) in the highest versus lowest quintiles of dietary intakes of vitamin B12 and methionine among alcohol never-drinkers were 2.82 (1.18–6.74; *p-*trend=0.009) and 3.45 (1.32–9.06; *p*-trend=0.003), respectively (Table 3). The HRs (95% CIs) in the highest versus lowest tertiles of dietary intakes of vitamin B12 and methionine among men who were never-drinkers were 2.92 (0.93–9.19; *p-*trend=0.02; *p*-interaction=0.24) and 3.30 (0.77–14.10; *p*-trend=0.004; *p*-interaction=0.03), respectively (Supplementary Table 1). There were no interactions by alcohol intake on the association of dietary intakes of vitamin B6 and folate with the risk of EC (Table 3).

**Table 3:** Hazard ratios (95% confident intervals) of esophageal cancer according to quintiles of energy-adjusted dietary intakes of vitamin B12, vitamin B6, folate, and methionine- stratified analysis by alcohol consumption

	Q1	Q2	Q3	Q4	Q5	***p***-for trend^**a**^	***p-***interaction
**Vitamin B12**							0.18
**Never drinker**							
Number at risk	9,333	9,956	9,899	9,591	9,224		
Person-years	160,871	168,690	165,816	159,579	153,650		
Case, n	7	11	19	26	23		
Model 2 ^b^	1.00 (reference)	1.45 (0.56–3.75)	2.31 (0.96–5.53)	3.27 (1.39–7.66)	2.82 (1.18–6.74)	0.009	
**Alcohol intake 0–150 g/week**							
Number at risk	2,554	3,142	3,425	3,697	3,380		
Person-years	44,250	53,644	57,610	61,874	56,835		
Case, n	8	7	12	15	16		
Model 2 ^b^	1.00 (reference)	0.70 (0.25–1.94)	1.08 (0.44–2.65)	1.21 (0.50–2.91)	1.28 (0.53–3.09)	0.34	
**Alcohol intake > 150 g/week**							
Number at risk	5,161	3,878	3,665	3,721	4,372		
Person-years	85,509	63,418	60,921	61,546	71,490		
Case, n	65	52	48	45	64		
Model 2 ^b^	1.00 (reference)	1.13 (0.78–1.64)	1.09 (0.74–1.61)	0.99 (0.66–1.47)	1.13 (0.78–1.63)	0.66	
**Vitamin B6**							0.89
**Never drinker**							
Number at risk	9,094	9,602	9,631	9,447	9,150		
Person-years	173,187	181,266	169,377	151,569	133,206		
Case, n	10	16	20	21	19		
Model 2 ^b^	1.00 (reference)	1.37 (0.62–3.03)	1.58 (0.73–3.39)	1.69 (0.78–3.64)	1.53 (0.69–3.37)	0.31	
**Alcohol intake 0–150 g/week**							
Number at risk	2,627	3,108	3,494	3,622	3,347		
Person-years	45,054	53,227	59,136	61,086	55,710		
Case, n	4	12	15	18	9		
Model 2 ^b^	1.00 (reference)	2.23 (0.72–6.94)	2.17 (0.72–6.60)	2.26 (0.75–6.80)	1.11 (0.33–3.68)	0.60	
**Alcohol intake > 150 g/week**							
Number at risk	4,478	3,205	3,385	4,236	5,493		
Person-years	73,024	53,030	56,065	70,334	90,430		
Case, n	61	47	46	43	77		
Model 2 ^b^	1.00 (reference)	1.10 (0.75–1.63)	1.00 (0.67–1.48)	0.79 (0.52–1.18)	1.03 (0.72–1.47)	0.83	
**Folate**							0.41
**Never drinker**							
Number at risk	7,560	9,277	9,946	10,401	10,819		
Person-years	129,722	156,106	166,190	175,262	181464		
Case, n	13	12	19	27	15		
Model 2 ^b^	1.00 (reference)	0.74 (0.33–1.69)	1.19 (0.57–2.50)	1.43 (0.71–2.92)	0.76 (0.35–1.69)	0.80	
**Alcohol intake 0–150 g/week**							
Number at risk	2,662	3,339	3,454	3,397	3,346		
Person-years	45,040	56,139	58,275	57,803	56,956		
Case, n	10	9	13	20	6		
Model 2 ^b^	1.00 (reference)	0.65 (0.26–1.61)	0.93 (0.40–2.14)	1.40 (0.64–3.05)	0.40 (0.14–1.12)	0.30	
**Alcohol intake > 150 g/week**							
Number at risk	6,891	4,389	3,546	3,183	2,788		
Person-years	112,892	72,205	58,818	52,931	46,036		
Case, n	84	58	44	42	46		
Model 2 ^b^	1.00 (reference)	1.12 (0.79–1.57)	1.06 (0.73–1.54)	1.05 (0.72–1.55)	1.30 (0.89–1.89)	0.25	
**Methionine**							0.02
**Never drinker**							
Number at risk	8,214	10,251	10,241	9,929	9,368		
Person-years	141,928	174,731	172,663	164,817	154,605		
Case, n	5	13	11	25	32		
Model 2 ^b^	1.00 (reference)	2.02 (0.72–5.68)	1.52 (0.52–4.38)	2.92 (1.10–7.71)	3.45 (1.32–9.06)	0.003	
**Alcohol intake 0–150 g/week**							
Number at risk	2,031	2,743	3,445	4,001	3,978		
Person-years	34,893	47,437	57,881	67,544	66,457		
Case, n	4	10	14	16	14		
Model 2 ^b^	1.00 (reference)	1.68 (0.53–5.37)	1.54 (0.50–4.74)	1.37 (0.45–4.14)	1.06 (0.34–3.27)	0.52	
**Alcohol intake > 150 g/week**							
Number at risk	6,852	3,956	3,309	3,067	3,613		
Person-years	112,220	65,083	54,758	51,056	59,764		
Case, n	98	65	28	32	51		
Model 2 ^b^	1.00 (reference)	1.23 (0.89–1.70)	0.64 (0.42–0.99)	0.80 (0.53–1.22)	1.03 (0.72–1.48)	0.68	

Abbreviations: HR, hazard ratio; 95% CI, 95% confidence interval.

^a^ Median values of vitamin B12, vitamin B6, folate, and methionine in each quintile were used to test for a linear trend across quintiles.

^b^ Model 2 was adjusted for age, sex, public health center area, body mass index (quintiles), smoking (never, past, current; ≤19 cigarettes/day, 20–29 cigarettes/day, or ≥30 cigarettes/day), alcohol consumption (non-drinkers, ≤150 g/week, 150<alcohol≤300 g/week, 300<alcohol<450 g/week, or ≥450 g/week) for the ever-drinkers’ group, family history of cancer, and physical activity in METs (quintiles)

## Discussion

In this large prospective study of Japanese men and women, we found that a higher dietary intake of vitamin B12 was associated with an increased risk of EC after adjusting for EC risk factors. When stratified by alcohol drinking status, we found positive associations of vitamin B12 and methionine with the risk of EC among never-drinkers but not among drinkers, either low or high intake alcohol drinkers. The other investigated nutrients, vitamin B6 and folate, were not associated with the risk of EC.

### Dietary intake of vitamin B12

The positive association between the dietary intake of vitamin B12 and the risk of EC was consistent with previous studies conducted in the USA and Europe [15, 16]. One of the studies that found a positive association between vitamin B12 intake and EC risk was a case-control study in the USA, which included 687 controls, 282 cases of esophageal adenocarcinoma, and 206 cases of esophageal squamous cell carcinoma. They found that the odds ratio (OR) (95% CI) of dietary vitamin B12 intake (comparing ≥75^th^ percentile of intake to 25^th^ percentile of intake) in esophageal adenocarcinoma versus control was 1.39 (1.10–1.76) and that in esophageal squamous cell carcinoma versus control was 1.51 (1.15–2.00) [15]. Another study that found a positive association between dietary intake of vitamin B12 and the risk of EC was a case-control study within the FINBAR study in Ireland that included 227 cases of esophageal adenocarcinoma and 260 controls. In that study, dietary vitamin B12 intake was positively associated with esophageal adenocarcinoma risk; the multivariable OR (95% CI) in the highest (≥9.7 μg/day) versus lowest (≤4 μg/day) quartiles of dietary vitamin B12 intake was 3.87 (2.22–6.73; *p-*trend was ≤0.01) [16]. The mechanism of the positive association between the dietary intake of vitamin B12 and risk of EC is still unclear. In the previous studies, the observed positive association were mainly attributed to the fact that the primary sources of vitamin B12 were animal origin foods, such as meat or fish [16, 29]. Patients with esophageal adenocarcinoma versus controls in the FINBAR study in Europe were more likely to consume higher amounts of fresh red meat; the multivariate OR (95% CI) was 3.15 (1.38–7.20) [30]. In Japan, the main dietary sources of vitamin B12 have been shown to be fish and shellfish, followed by meat [31]. We analyzed the data of our study to examine the association of fish and shellfish, and total meat intake with EC risk; however, we did not find any significant association for either food. We also examined the association between the intake of dairy products and eggs, other food sources of vitamin B12, and the risk of EC, and we found no association between them (not shown in table). Furthermore, there was no significant change in the association between vitamin B12 and the risk of EC in the fully-adjusted model that included fish and shellfish, total meat, eggs, and dairy products (HR and 95%CI 1.73[1.11-2.68], *p*-trend=0.01) (not shown in table). A molecular study found that aberrant DNA methylation might be involved in the pathogenesis of esophageal squamous cell carcinoma [32]. In addition, a meta-analysis study showed that one-carbon metabolism nutrients were associated with aberrant DNA methylation [33]. Despite that, the complex nature of the overall dietary patterns and disease risk association suggests that the positive association between high vitamin B12 intake and the risk of EC might reflect that vitamin B12 is a surrogate of pro-carcinogenic diet or vitamin B12 *per se* may increase the cancer risk; however, further research need to be conducted to investigate this hypothesis.

One carbon-metabolism nutrients act interactively to affect health and cancer pathogenesis [34]. Therefore, to control the interactions between B vitamins in this study, we conducted the analysis with a mutually adjusted model that included all of the B vitamins and methionine simultaneously (Model 3). We observed that the positive association between vitamin B12 and the risk of EC became stronger after controlling for other studied B vitamins and methionine.

The positive association between vitamin B12 intake and EC risk was only found among participants who were not alcohol-drinker. However, *p*-interaction with alcohol intake did not reach statistical significance (*p*-interaction =0.18). A similar finding was also observed in men; however, we could not conduct the stratified analysis based on alcohol intake in women due to the small number of cases. Biologically, alcohol could cause malabsorption of vitamin B12 and decrease its bioavailability [21, 35]. A randomized controlled trial showed a 5% decrease in blood vitamin B12 levels in postmenopausal women who had been given 15 g and 30 g alcohol for 8 weeks compared with subjects who had not been given alcohol [21]. Consistent with this finding, another randomized-controlled trial found a 6% decrease in plasma vitamin B12 levels in male volunteers after a 2-week red wine and vodka intervention [35]. The positive association between higher dietary intake of vitamin B12 and EC risk that was only found among alcohol never-drinkers may be because there is no reduction in vitamin B12 bioavailability in the bodies of these individuals. The cancer-promoting effect of vitamin B12 may be weaker than that of alcohol, a strong risk factor for EC [36, 37]. Therefore, this could have also been the reason for not detecting the effect of vitamin B12 on EC risk in low and high alcohol drinkers.

### Dietary intake of methionine

There was no association found between dietary methionine intake and EC risk. In the stratified analysis based on alcohol consumption, we found a significant positive association between dietary methionine intake and EC risk among never-drinkers. This positive association was similar to that of vitamin B12. In fact, there was a high Pearson correlation coefficient between dietary intakes of methionine and vitamin B12 (r=0.74; *p*<0.0001).

Biologically, vitamin B12 is an essential substrate of the methionine cycle in the OCM pathway [38]. An animal study showed a decrease in methionine uptake in the intestines of rats after 40 days of alcohol intake [39]. Thus, an effect of methionine on the risk of EC was expected among never-drinkers.

### Dietary intake of folate and vitamin B6

There was no association between dietary intake of folate and the risk of EC. Our finding is supported by a cohort study from Uruguay, which found that the multivariable HR (95% CI) of EC in the highest versus lowest quartiles of dietary folate intake was 1.15 (0.83–1.59; *p*=0.34) [40]. On the other hand, several other studies reported an inverse association between dietary intake of folate and risk of EC, even after multivariable adjustment.[14–19, 41] The biological justification for this discrepancy is currently lacking; however, in the studies that found an inverse association between dietary folate intake and EC risk, the median folate intake in the highest quintile or quartile was lower than that in our study: 638 μg/day in our study versus 379 μg/day and 275 μg/day in the studies by Ibiebele et al. and Aune et al., respectively [14, 18]. The NIH-AARP Diet and Health Study from the USA, which had a similar high intake of folate (median intake values of 288 mcg/day in Q1 up to 566 mcg/day in Q5) and used Q3 (405 mcg/day) as the reference category, found an increased risk of esophageal squamous cell carcinoma with lower intake of folate: HR (95% CI) for Q1 versus Q3 was 1.91 (1.17–3.10); however, no reduced risk was found with higher folate intake: HR (95% CI) for Q5 versus Q3 was 1.07 (0.59–1.94) [19]. Although the FFQ was not able to measure absolute nutrient intake and thus, did not allow its comparison with the RDA, it is worth noting that the dietary folate intake in Q5 in our study was higher than that in the Japanese RDA, even though Japan does not have mandatory folic acid food fortification policy [42]. Our study may be in line with the NIH-AARP Diet and Health Study that found no association between folate intake and EC risk among those with a higher dietary folate intake [19].

There was no association between the dietary intake of vitamin B6 and risk of EC, which was consistent with the results of a cohort study from the USA [19]. However, a case-control study from Ireland by Sharp et al. reported an inverse association between vitamin B6 intake and EC risk; the OR (95% CI) in the highest versus lowest quartiles of vitamin B6 intake was 0.37 (0.22–0.63; *p*-trend was <0.01) [16]. This discrepancy may be due to the different adjustment factors that were used. Compared with their study that did not include smoking status, our study included smoking status in the analyses [16]. It is worth noting that Sharp et al. found inverse associations of dietary intake of folate and vitamin B6 only with the risk of esophageal adenocarcinoma as they did not have cases of esophageal squamous cell carcinoma [16]. In our study, the dominant histological EC subtype was esophageal squamous cell carcinoma. An Australian case-control study that included 364 esophageal adenocarcinoma and 306 esophageal squamous cell carcinoma cases reported inverse associations between folate and vitamin B6 intakes and esophageal adenocarcinoma risk. For esophageal adenocarcinoma, the multivariable HRs (95% CIs) in the highest versus lowest quartiles of intakes were 0.72 (0.53–0.98; *p*-trend=0.01) and 0.53 (0.39–0.74; *p*-trend=0.002) for folate and vitamin B6, respectively; however, no associations with the risk of esophageal squamous cell carcinoma were found; HRs were 0.78 (0.51–1.19; *p*-trend=0.06) and 0.66 (0.42–1.05; *p*-trend=0.08) for dietary intakes of folate and vitamin B6, respectively [18].

In our study, the dominant histological EC subtype was squamous cell carcinoma, and it mostly occurred in men; this was consistent with previous findings in Japan that esophageal squamous cell carcinoma accounted for 89.5% of all EC cases, and 84.9% of these esophageal squamous cell carcinoma cases occurred in men [43]. Due to the small number of esophageal adenocarcinoma cases, we could not conduct a stratified analysis based on histological subtype. However, when we limited the analysis to esophageal squamous cell carcinoma, there was an increased risk of esophageal squamous cell carcinoma in men with higher dietary intakes of vitamin B12 (data not shown).

### Strengths and limitations

This was the first large prospective cohort study to investigate the association of B vitamins and methionine intake with EC risk in Japan. Our study had several strengths, including a long follow-up time, validated dietary intakes, and a large sample size, which allowed the stratified analyses based on alcohol intake to detect the possible effect modification by alcohol consumption.

Nevertheless, this study also had some limitations. First of all, the blood biomarkers of vitamin B12 status were not assessed. According to previous research from the JPHC study, the blood measurement of vitamin B12 status was only conducted in less than 1 % of the JPHC population. Furthermore, the observed correlation coefficients between dietary intakes of vitamin B12, vitamin B6, and folate with the blood concentrations of these vitamins were relatively low, especially for vitamin B12; Spearman correlations value were 0.06, 0.23, and 0.23, respectively [44]. Dietary intake of vitamin B12 is a relatively poor reflection of vitamin B12 status because the bioavailability of vitamin B12 may be affected by individual variance in vitamin B12 absorption [45]. For example, malabsorption of vitamin B12 could be caused by chronic gastric inflammation caused by atrophic gastritis, inflammatory bowel disease, or prolonged use of drugs that affect the pH of the stomach such as proton-pump inhibitors and H2-receptor antagonists, and metformin by affecting the vitamin B12 receptor [46–49].

Second, the most dominant histological EC subtype was squamous cell carcinoma, and there were only a few esophageal adenocarcinoma cases (*n*=17); thus, we could not evaluate the associations of B vitamins and methionine intake with adenocarcinoma EC subtype risk specifically.

Third, we lacked information on the use of vitamin B supplementation; however, the use of vitamin supplements during the time of the baseline survey (1990s) was not common in Japan. Finally, information about gastroesophageal reflux disease or Barrett’s esophagus as strong risk factors for EC was unavailable.

## Conclusions

Our study showed that a higher dietary intake of vitamin B12 was associated with an increased risk of EC. In the stratified analysis by alcohol intake status, the positive associations of vitamin B12 and methionine intake with the risk of EC were found among never-drinkers. On the other hand, there was no significant association between vitamin B12 and methionine intake and the risk of EC among both low-level (0-150 g/week) and high-level (>150 g/week) alcohol drinkers. Future research is warranted on the association between vitamin B12 and the risk of EC in other populations.

## Supplementary Information

**Additional file 1.**

## Data Availability

The datasets generated and/or analysed during the current study are not publicly available due to participant privacy, according to ethical guidelines in Japan. Additionally, the informed consent we obtained does not include a provision for publicly sharing data. The data are available from the JPHC Study Group at https://epi.ncc.go.jp/en/jphc/805/8155.html or the Office of The JPHC Study Group at jphcadmin@ml.res.ncc.go.jp on reasonable request.

## Abbreviations

BMI: Body mass index
CI: Confidence interval
EC: Esophageal cancer
FFQ: Food Frequency Questionnaire
HR: Hazard ratio
JPHC: Japan Public Health Center-Based Prospective Study
MET: Metabolic equivalent of task
OCM: One-carbon metabolism
OR: Odds ratio
PHC: Public health center
RDA: Recommended Dietary Allowance
