# Supplementary material for: Association of dietary intakes of vitamin B12, vitamin B6, folate, and methionine with the risk of esophageal cancer: the Japan Public Health Center-based (JPHC) prospective study
Source: BMC Cancer. 2021 Sep 1;21:982. doi: 10.1186/s12885-021-08721-8 (PMC8411535; doi:10.1186/s12885-021-08721-8)
Supplement: Supplementary file 1 — Additional file 1. [file 12885_2021_8721_MOESM1_ESM.rtf]

Supplementary Table 1. Hazard ratios (95% confident intervals) of esophageal cancer according to quintiles of energy-adjusted dietary intakes of vitamin B12 and methionine- stratified analysis by alcohol intake in men 

�@	Q1	Q2	Q2	Q4	Q5	p for trend a	p interaction	
Vitamin B12	�@	�@	�@	�@	�@	�@	0.24	
Never drinker	
Number at risk	1,596	1,934	2,306	2,417	2,525	�@	�@	
Person-years	26,295	30,347	35,555	36,133	37,674	�@	�@	
Case, n	4	4	13	18	14	�@	�@	
Model 2 b	1.00 (reference)	0.90 (0.22 -3.61)	2.49 (0.81 -7.69)	3.72 (1.24-11.19)	2.92 (0.93 -9.19)	0.02	�@	
Alcohol intake 0–150 g/week	
Number at risk	1,470	1,849	2,095	2,226	2,101		�@	
Person-years	24,949	31,117	34,389	36,403	34,084		�@	
Case, n	8	6	10	12	14		�@	
Model 2 b	1.00 (reference)	0.59 (0.20 -1.69)	0.85 (0.33 -2.17)	0.91 (0.36 -2.29)	1.03 (0.42 -2.54)	0.62		
Alcohol intake > 150 g/week	
Number at risk	4,871	3,675	3,459	3,512	4,123			
Person-years	80,664	60,168	57,402	58,045	67,251			
Case, n	65	50	48	44	64			
Model 2 b	1.00 (reference)	1.09 (0.75 -1.59)	1.10 (0.74 -1.62)	0.97 (0.65 -1.45)	1.13 (0.78 -1.64)	0.62		
Methionine	�@	�@	�@	�@	�@	�@	0.03	
Never drinker	
Number at risk	821	1,249	1,960	2,947	3,801		�@	
Person-years	13,583	19,965	30,346	44,563	57,548		�@	
Case, n	2	1	7	18	25		�@	
Model 2 b	1.00 (reference)	0.35 (0.03 -3.89)	1.62 (0.34 -7.86)	2.81 (0.65 -12.23)	3.30 (0.77 -14.10)	0.004	�@	
Alcohol intake 0–150 g/week	
Number at risk	758	1,243	2,002	2,720	3,018			
Person-years	12,532	21,000	33,137	44,896	49,376			
Case, n	2	9	12	13	14			
Model 2 b	1.00 (reference)	2.77 (0.60- 12.88)	2.14 (0.47 -9.61)	1.74 (0.39 -7.76)	1.56 (0.35 -6.92)	0.59		
Alcohol intake > 150 g/week	
Number at risk	6,314	3,734	3,144	2,927	3,521		�@	
Person-years	103,447	6,135	51,908	48,663	58,157		�@	
Case, n	96	65	27	32	51		�@	
Model 2 b	1.00 (reference)	1.25 (0.91 -1.74)	0.63 (0.41 -0.98)	0.82 (0.54 -1.25)	1.05 (0.73 -1.51)	0.71	�@	

Abbreviations: HR, hazard ratio; 95% CI, 95% confidence interval.	�@	�@	�@
a Median values of vitamin B12 and methionine in each quintile were used to test for a linear trend across quintiles.
b Model 2 was adjusted for age, public health center area, body mass index (quintiles), smoking (never, past, current; ≤19 cigarettes/day, 20–29 cigarettes/day, ≥30 cigarettes/day) , alcohol consumption (non-drinkers, ≤150 g/week, 150<alcohol≤300 g/week, 300<alcohol<450 g/week, ≥450 alcohol g/week for the ever-drinkers' group), family history of cancer, and physical activity in METs (quintiles)
